# Engineered celastrol liposomes with glycyrrhizic acid augment synergistic antitumor efficacy in breast cancer

**DOI:** 10.3389/fonc.2025.1602585

**Published:** 2025-05-20

**Authors:** Wei Zhang, Jiping Li, Xiuli Gao, Likun Liu, Hong Liang, Liling Yue

**Affiliations:** ^1^ Office of Academic Research, Qiqihar Medical University, Qiqihar, China; ^2^ School of Public Health, Qiqihar Medical University, Qiqihar, China; ^3^ Research Institute of Medicine of Pharmacy, Qiqihar Medical University, Qiqihar, China

**Keywords:** celastrol, glycyrrhizic acid, liposomes, breast cancer, DNA damage

## Abstract

**Background/Objectives:**

Breast cancer treatment remains challenged by the non-specific distribution and systemic toxicity of conventional chemotherapeutics. Celastrol (CEL), a natural compound with antitumor efficacy, faces clinical limitations due to its toxicity and poor solubility. Glycyrrhizic acid (GA), known for tumor-suppressive and membrane-modifying properties, offers potential to enhance targeted drug delivery. This study aimed to develop GA-modified liposomes (GA/LIP-CEL) to synergistically improve CEL’s therapeutic specificity and safety.

**Methods:**

GA/LIP-CEL nanoparticles were engineered via thin-film hydration, replacing cholesterol with GA. Physicochemical properties were characterized using TEM, DLS, and HPLC. *In vitro* evaluations included stability assays, cellular uptake (flow cytometry), cytotoxicity (MTT assay), mitochondrial membrane potential (JC-1 staining), apoptosis (Annexin V/PI), DNA damage (γ-H2AX immunofluorescence), and cell cycle analysis in BT549 breast cancer and MCF-10A normal cells.

**Results:**

GA/LIP-CEL exhibited uniform spherical morphology (122.48 ± 5.37 nm), high drug loading (87.75 ± 2.61%), sustained release (70.13% cumulative release at 24 h), and colloidal stability (negligible size variation over 14 days). Compared to LIP-CEL, GA/LIP-CEL reduced IC50 in BT549 cells while lowering cytotoxicity in MCF-10A cells. Compared with the LIP-CEL group, GA/LIP-CEL treatment demonstrated a statistically significant increase in apoptotic cell proportion (P < 0.05). Enhanced mitochondrial dysfunction (P < 0.05) and DNA (P < 0.05) damage were observed. GA modification improved cellular uptake potentially via regulating membrane fluidity and receptor-mediated endocytosis effects, and induced S-phase arrest (31.66 ± 1.70% cells).

**Conclusions:**

GA/LIP-CEL combines GA’s membrane-targeting capabilities with CEL’s therapeutic effects, improving stability, specificity, and safety. This platform represents a novel strategy for precision drug delivery, addressing limitations of conventional systems through natural component integration. Further validation of *in vivo* performance and pharmacokinetics is warranted to advance clinical translation.

## 1 Introduction

Breast cancer has remained the most prevalent malignancy among women worldwide. Although significant progress had been achieved through conventional therapies including surgery, chemotherapy, and targeted treatments, major clinical challenges persisted due to the non-specific distribution of chemotherapeutic agents, systemic toxicity, and tumor microenvironment-mediated drug resistance (1). Recent advancements in targeted therapies, particularly CDK4/6 inhibitors, were demonstrated to significantly improve patient survival rates, though their long-term application was constrained by hematologic toxicity and gastrointestinal adverse effects (2, 3). Furthermore, tumor heterogeneity and metastasis recurrence were identified as critical unresolved issues, necessitating the development of innovative therapeutic strategies that combined high efficacy, low toxicity, and precise targeting of the tumor microenvironment (4).

Recent years have witnessed significant advancements in nano-engineering of natural bioactive compounds for breast cancer intervention (5). Celastrol (CEL), a triterpenoid isolated from Tripterygium wilfordii, was demonstrated to exert potent anti-tumor effects through cell cycle arrest induction, survivin suppression, and mitochondrial apoptosis activation (6, 7). However, its clinical application was significantly hindered by poor aqueous solubility and nonspecific distribution, which lead to systemic toxicity, such as liver and kidney damage (8).

Glycyrrhizic acid (GA), an amphiphilic triterpene saponin, was validated to possess hepatoprotective, anti-inflammatory, and tumor-suppressive properties (9). Its chemoprotective potential was further evidenced by attenuation of chemotherapeutic drugs-induced hepatotoxicity and nephrotoxicity in preclinical studies (10). The unique molecular architecture of GA, characterized by hydrophilic glucuronic acid moieties and hydrophobic aglycone domains, enabled effective lipid bilayer integration, enhancing nanocarrier stability and tumor accumulation (10–12). As a natural amphiphilic functional molecule, GA has been widely utilized in the construction of nano-drug delivery systems. Studies have demonstrated that GA can form stable complexes with hydrophobic bioactive components such as berberine, baicalein, tanshinone IIA, hydroxycamptothecin, and curcumin. Through nano-formulation strategies, these complexes significantly reduce drug toxicity, enhance solubility and physicochemical stability, elevate plasma concentration, improve bioavailability, and augment tumor-targeting efficiency (13). Notably, the synergistic enhancement of therapeutic efficacy by GA-modified nanosystems may be attributed to GA receptor-mediated endocytosis effects and GA-regulated membrane fluidity (14). Synergistically, GA-mediated tumor microenvironment remodeling was observed to suppress P-glycoprotein efflux activity and reverse multidrug resistance, providing new ideas for combination therapy (15, 16).

**Scheme 1:**
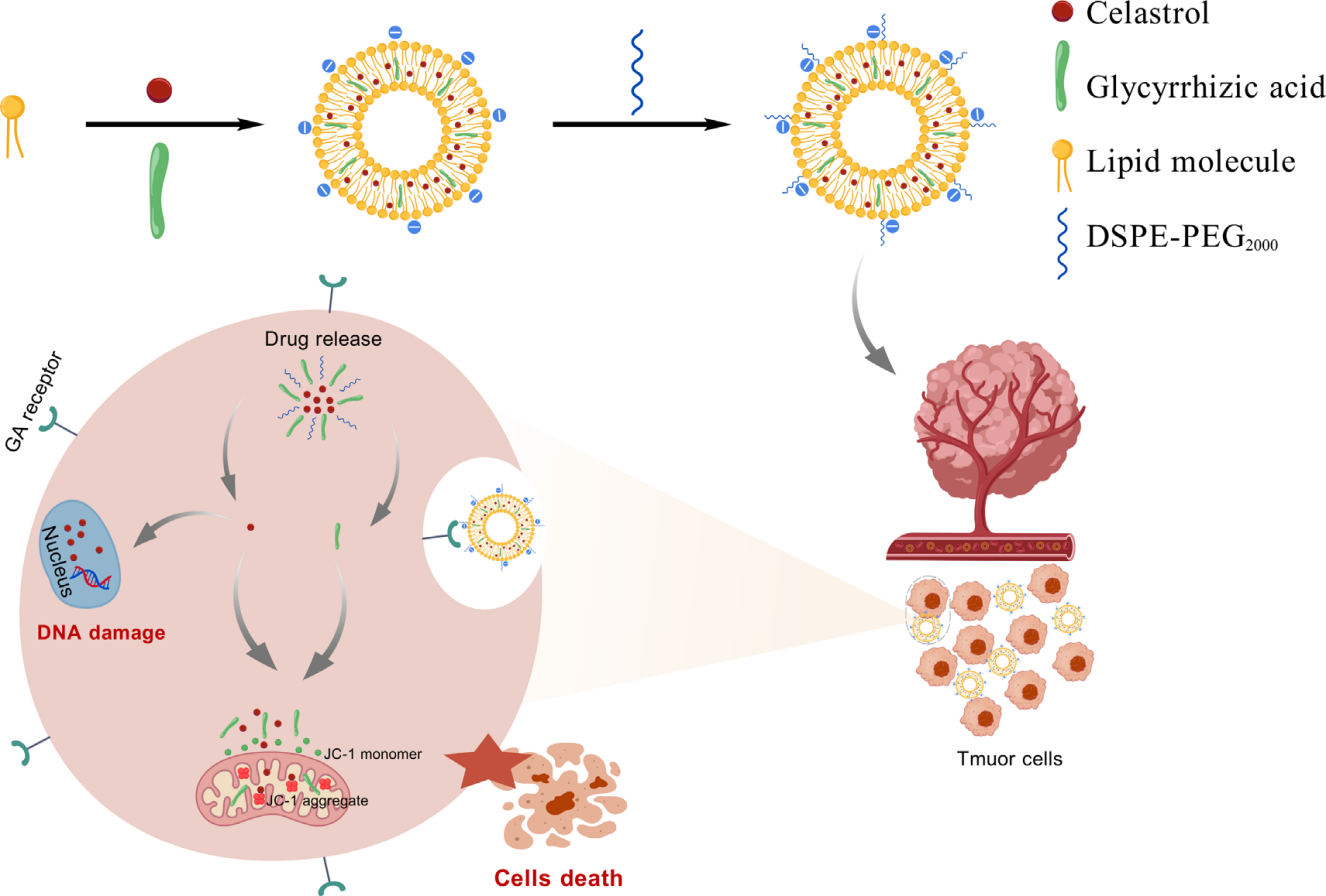
Graphic illustration of the preparation strategy and the antitumor function of GA/LIP-CEL nanoparticles.

While nanodelivery systems (e.g., liposomes, inorganic nanoparticles) were known to improve drug distribution through the enhanced permeability and retention (EPR) effect, conventional nanocarriers continued to face limitations including low targeting efficiency, rapid immune clearance, and insufficient drug-loading stability (17). This study aimed to develop a glycyrrhizic acid-functionalized liposomal platform (GA/LIP-CEL) co-encapsulating CEL to address its toxicity profile while enhancing tumor specificity. GA’s amphiphilic properties enable structural optimization through modulation of lipid bilayer packing density, improving drug-loading stability and tumor-selective uptake As showed in **Scheme 1** (created with BioGDP.com), the therapeutic efficacy originated from sustained CEL release-triggered DNA damage response via double-strand DNA break induction, combined with GA-mediated mitochondrial membrane potential depolarization to cooperatively activate intrinsic apoptosis pathways in breast carcinoma cells. Furthermore, the therapeutic strategy leveraged GA-mediated endocytosis coupled with CEL’s multi-target antitumor mechanisms, establishing a synergistic therapeutic paradigm to achieve multi - pathway antitumor synergy.

## 2 Results and discussion

### 2.1 Characterization and properties of GA/LIP-CEL

**Figure 1:**
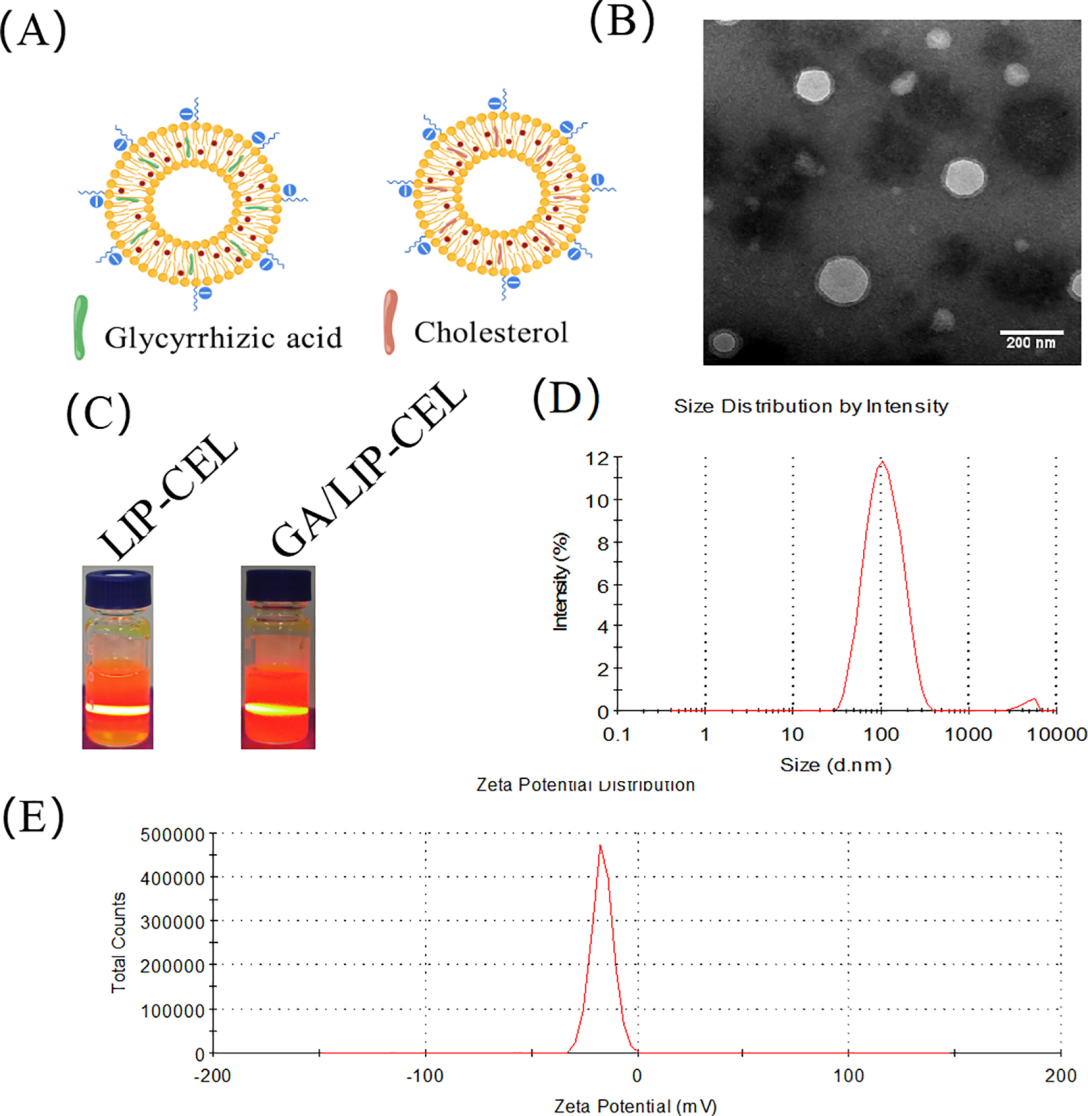
Characterization of GA/LIP-CEL. **(A)** Schematic illustration of LIP-CEL and GA/LIP-CEL. **(B)** Representative TEM images and photographs of GA/LIP-CEL. **(C)** Photographs of LIP-CEL and GA/LIP-CEL. **(D)** Hydrodynamic size of GA/LIP-CELL. **(E)** Zeta potential of GA/LIP-CEL.

LIP-CEL and GA/LIP-CEL were successfully prepared using the thin-film hydration method, forming uniform vesicular structures (**Figure 1A**). TEM revealed spherical nanoparticles with smooth surfaces for GA/LIP-CEL (**Figure 1B**), consistent with conventional cholesterol-containing liposomes, indicating that GA substitution did not significantly alter morphological integrity. The colloidal stability of both systems was confirmed by visible Tyndall effects under light scattering (**Figure 1C**).

Dynamic light scattering (DLS) measurements showed average hydrodynamic diameters of 111.88 ± 4.91 nm for LIP-CEL and 122.48 ± 5.37 nm for GA/LIP-CEL (**Figure 1D**, **Table 1**). Encapsulation efficiency (EE) values were 81.77 ± 3.19% for LIP-CEL and 87.75 ± 2.61% for GA/LIP-CEL (**Table 1**). The marginal size increase in GA/LIP-CEL may be attributed to the unique spatial configuration of GA molecules, which likely enhanced drug embedding efficiency within the lipid bilayer. Both formulations exhibited negative zeta potentials of −21.75 ± 1.53 mV and −23.76 ± 3.39 mV (**Figure 1E**, **Table 1**). In colloidal dispersion systems, the elevated absolute value of zeta potential usually indicates improved colloidal stability and prolonged systemic circulation by reducing immune clearance, as supported by prior studies (18, 19). These physicochemical alterations imply that GA modulates phospholipid packing order and membrane fluidity, thereby influencing liposomal dimensions and surface characteristics.

**Table 1:** Characterization of LIP-CEL and GA/LIP-CEL (Mean ± SD, n = 3).

Samples	Particle size (nm)	PDI	Zeta potential (mV)	EE (%)	LE (%)
LIP-CEL	111.88 ± 4.91	0.21 ± 0.04	−21.75 ± 1.53	81.77 ± 3.19	4.66 ± 0.40
GA/LIP-CEL	122.48 ± 5.37	0.23 ± 0.02	−23.76 ± 3.39	87.75 ± 2.61	5.25 ± 0.29

### 2.2 Stability of GA/LIP-CEL

**Figure 2:**
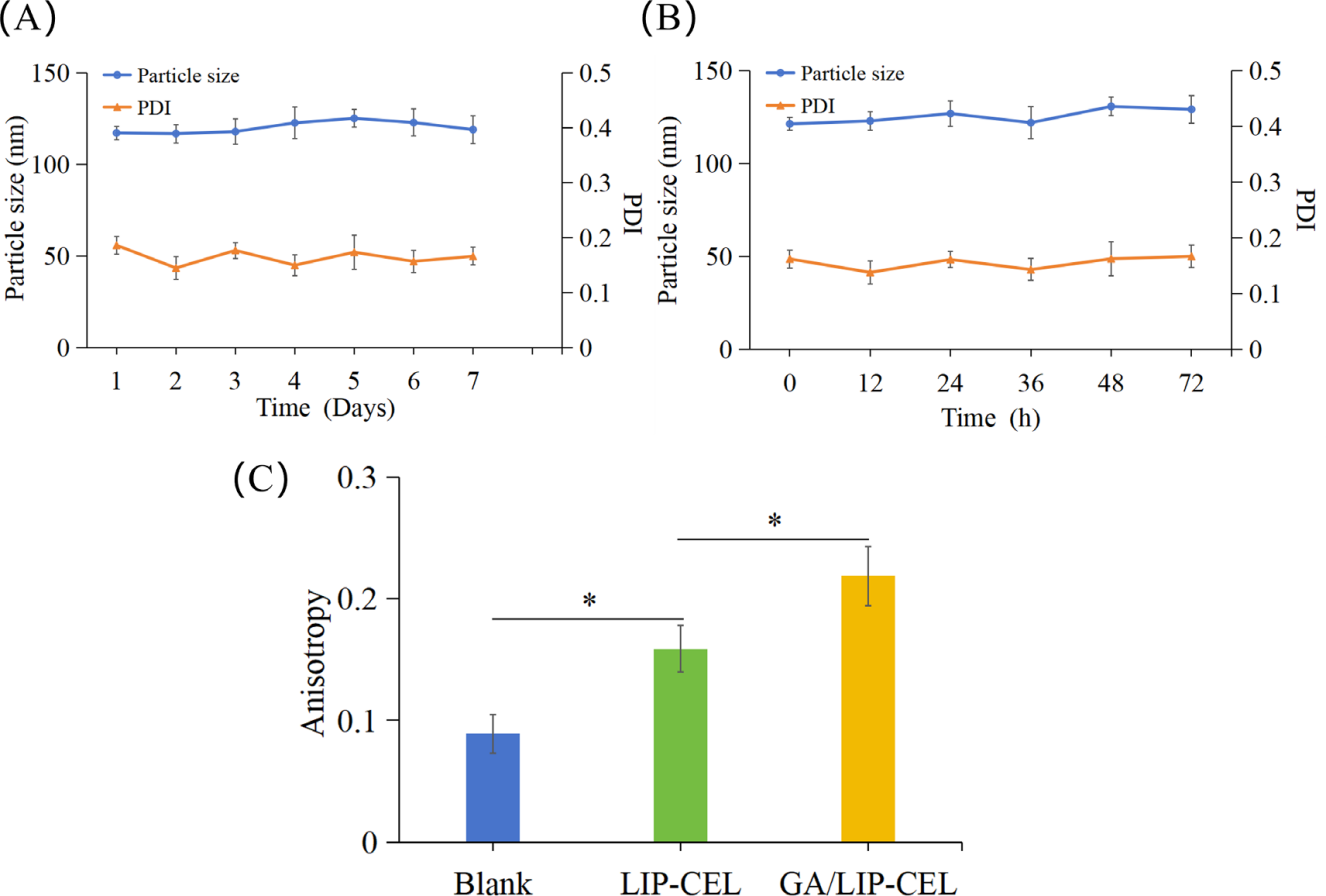
Stability of GA/LIP-CEL. **(A)** storage stability of GA/LIP-CEL within 7 days at 4°C. **(B)** Stability of GA/LIP-CEL incubated with 10% FBS at 37°C for 72 h. **(C)** Membrane fluidity of LIP-CEL and GA/LIP-CEL using a DPH fluorescent probe. * *P* < 0.05.

Liposomal stability is critical for achieving controlled drug release and minimizing systemic toxicity. The storage stability of GA/LIP-CEL was evaluated by monitoring particle size variations over 7 days at 4°C. Both formulations exhibited negligible size changes, confirming robust colloidal stability (**Figure 2A**). To assess physiological stability, nanoparticles were incubated with 10% FBS at 37°C for 72 h. Dynamic light scattering analysis revealed minimal size variations for both formulations (**Figure 2B**), demonstrating that GA effectively mimicked cholesterol’s membrane-stabilizing function.

To investigate the impact of GA substitution for cholesterol on liposomal membrane fluidity, the fluorescence anisotropy of various formulations was quantitatively assessed at 37°C using the DPH fluorescent probe labeling method. As illustrated in **Figure 2C**, Blank liposomes lacking cholesterol or GA exhibited the lowest fluorescence anisotropy value, whereas both LIP-CEL and GA/LIP-CEL formulations demonstrated elevated anisotropy levels (*P* < 0.05). These findings indicated that the incorporation of either cholesterol or GA significantly reduced lipid bilayer fluidity and permeability, thereby enhancing the nanoparticle stability. Notably, the GA/LIP-CEL system displayed a further increase in fluorescence anisotropy compared to LIP-CEL (*P* < 0.05), suggesting that GA’s triterpenoid saponin structure facilitated denser molecular packing within the lipid bilayer. Such structural optimization likely contributed to improved colloidal stability during storage and systemic circulation, providing experimental validation for GA as a cholesterol alternative in advanced delivery systems.

### 2.3 *In vitro* release of CEL from GA/LIP-CEL

**Figure 3:**
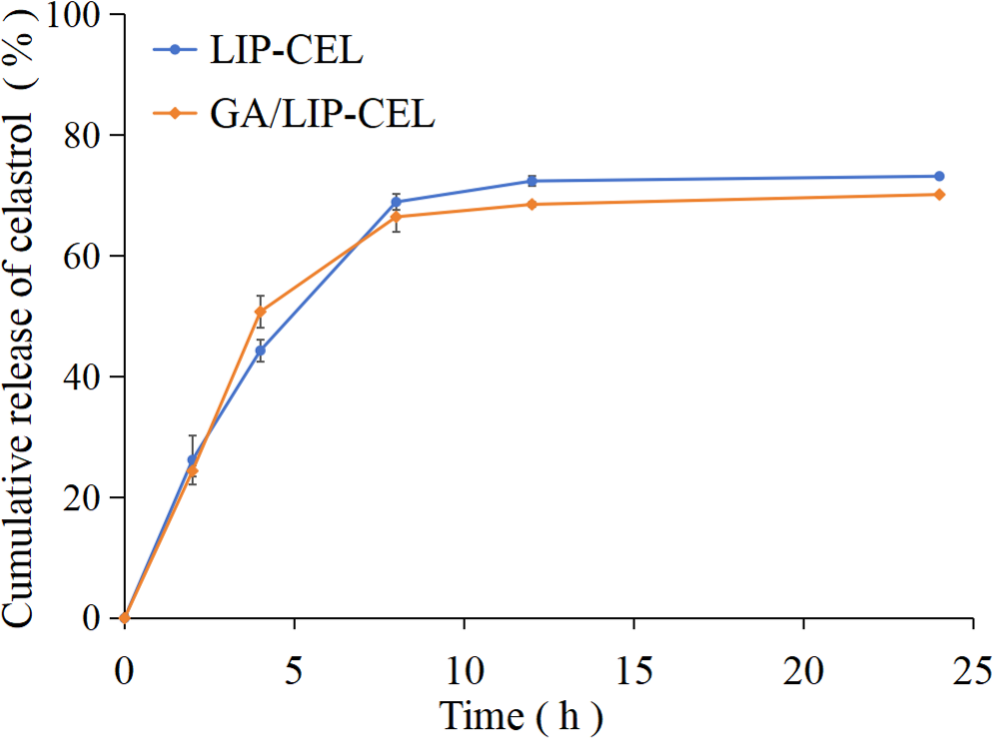
Time-dependent CEL release profile of LIP-CEL and GA/LIP-CEL. Data are shown as mean ± SD (n = 3).

The drug release profiles of LIP-CEL and GA/LIP-CEL were assessed via dialysis under physiological conditions. Both formulations demonstrated sustained release patterns, with GA/LIP-CEL exhibiting slower drug release profile than LIP-CEL from the initial 1-h timepoint (**Figure 3**). At 24 h, cumulative release rates reached 73.17% for LIP-CEL and 70.13% for GA/LIP-CEL. This phenomenon was attributed to GA-mediated modulation of lipid bilayer dynamics, where its molecular integration reduced membrane fluidity and permeability, thereby enhancing structural integrity (20). Furthermore, the hydrophilic GA coating likely acted as a steric barrier, impeding rapid drug diffusion while enabling controlled release through gradual hydration. These findings validate GA as a promising cholesterol alternative for developing low-toxicity, naturally derived liposomal systems with optimized encapsulation efficiency, stability, and tunable release kinetics.

### 2.4 Cellular uptake of GA/LIP-CEL in breast cancer cells

**Figure 4:**
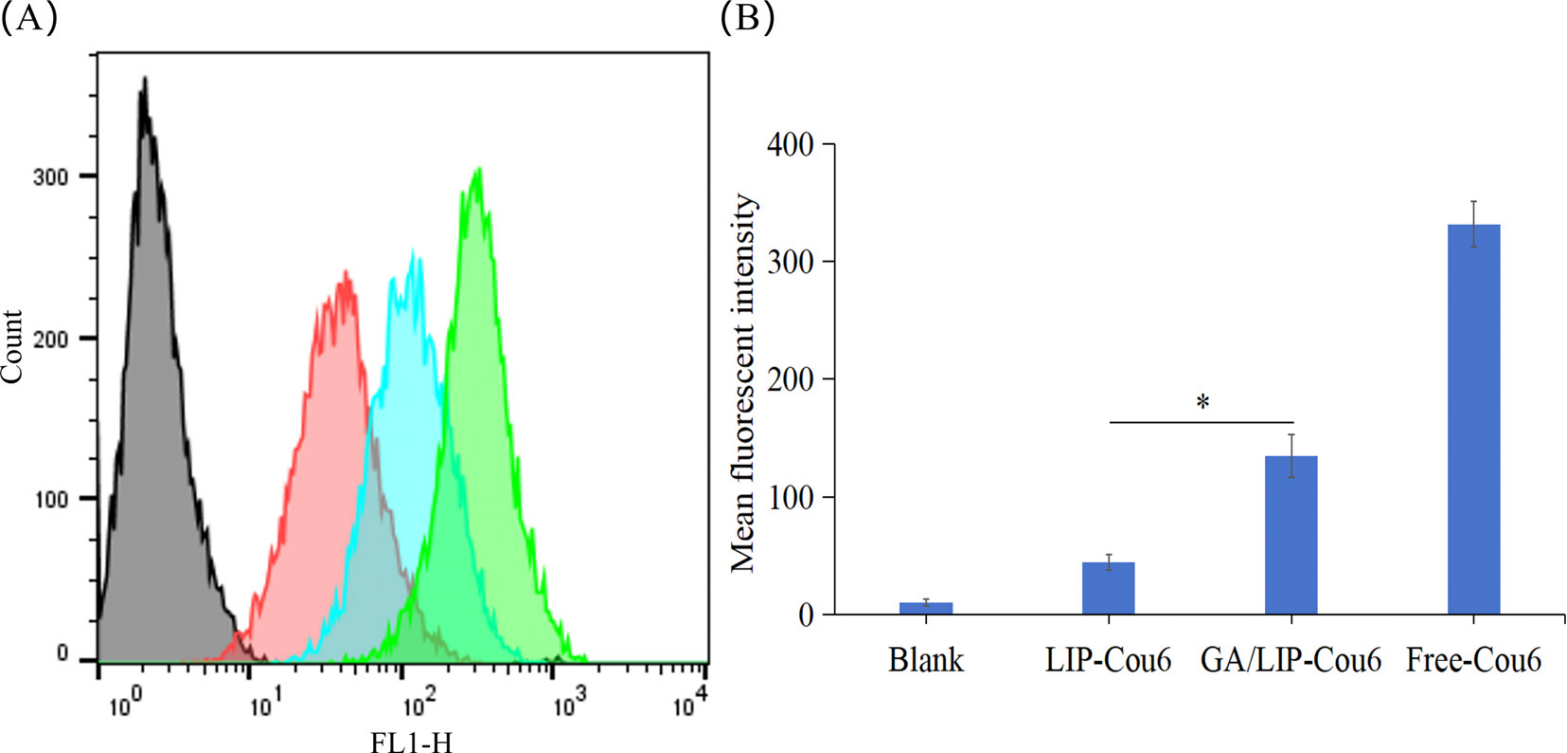
**(A)** Flow cytometric quantitative determination of Cou 6 uptake. **(B)** Quantitative analysis of Cou6 uptake based on flow cytometric plots. The results are shownas a mean fluorescence intensity ± SD (n = 3), * *P* < 0.05.

The enhanced cytotoxicity of nanoparticles toward cancer cells is generally attributed to improved endocytic efficiency (21). To evaluate the cellular uptake capacity of various formulations in breast cancer cells, BT549 cells were incubated with Free-Cou6, LIP-Cou6, and GA/LIP-Cou6 for 2 h, followed by flow cytometric quantification of cellular uptake. Notably, significantly higher intracellular fluorescence intensities were observed in cells treated with LIP-Cou6 and GA/LIP-Cou6 compared to Free-Cou6, with GA/LIP-Cou6 demonstrating the most pronounced fluorescence enhancement (*P* < 0.05) (**Figures 4A, B**), suggesting optimized cellular internalization of the GA-modified formulation. Collectively, these findings indicate that our engineered GA/LIP-Cou6 nanoparticles exhibit remarkable cellular uptake efficiency. The superior cellular uptake efficiency of GA/LIP-Cou6 may be attributed to GA-mediated receptor-dependent endocytosis effects and its regulated membrane fluidity. These results demonstrate that the engineered GA/LIP platform synergistically enhances cellular internalization through the dual mechanisms mentioned above, thus establishing a mechanistic basis for its therapeutic superiority in anticancer applications.

### 2.5 Inhibitory effect of GA/LIP-CEL on proliferation of breast cells

**Figure 5:**
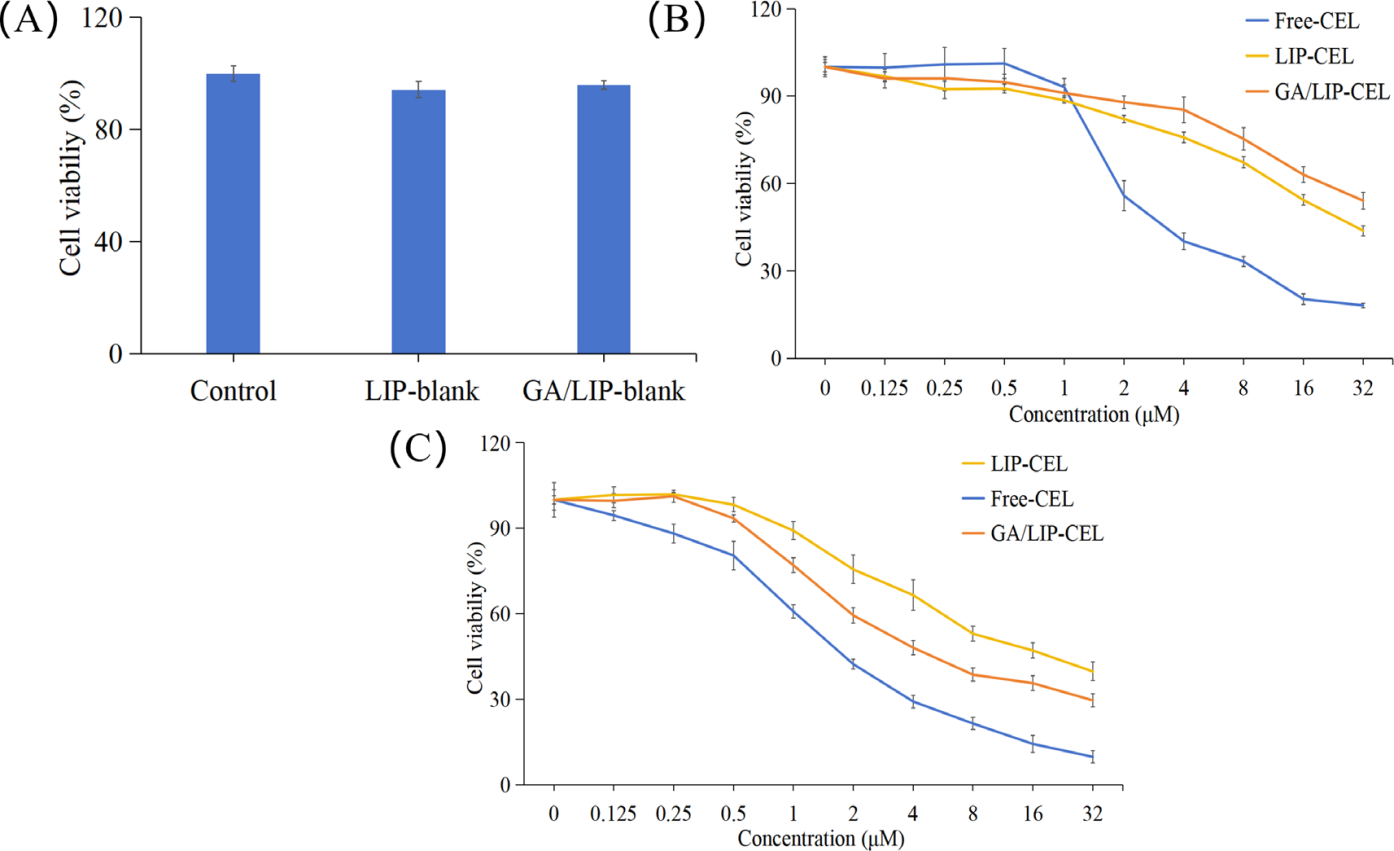
**(A)** *In vitro* cytotoxicity evaluation of LIP-blank and GA/LIP-blank in MCF-10A cells for 24 h. **(B)** *In vitro* cytotoxicity evaluation of LIP-CEL, GA/LIP-CEL and Free-CEL in MCF-10A cells for 24 h. **(C)** *In vitro* cytotoxicity evaluation of LIP-CEL, GA/LIP-CEL and Free-CEL in BT549 cells for 24 h. The data are presented as mean ± SD (n = 3).

The development of nanocarriers with appropriate biocompatibility and negligible cytotoxicity is paramount for their potential clinical applications in drug delivery (22, 23). In this study, preliminary cytotoxicity assessment of LIP-blank and GA/LIP-blank against human normal mammary epithelial cells (MCF-10A) was conducted using the methyl thiazolyl tetrazolium (MTT) assay. As shown in **Figure 5A**, both LIP-blank and GA/LIP-blank exhibited negligible cytotoxicity toward MCF-10A cell proliferation across all tested concentrations (*P* > 0.05). For drug-loaded formulations, Free-CEL, LIP-CEL, and GA/LIP-CEL were assessed in MCF-10A cells. Intriguingly, at equivalent concentrations, the LIP-CEL and GA/LIP-CEL groups demonstrated higher cell viability compared to the Free-CEL group, with GA/LIP-CEL showing the most pronounced preservation of normal cell viability (**Figure 5B**). These results indicate that GA modification effectively mitigates CEL-induced cytotoxicity toward healthy cells.

In breast cancer cells, all formulations inhibited proliferation in concentration- and time-dependent manners (**Figure 5C**). Free-CEL markedly suppressed breast cancer proliferation, particularly at higher concentrations. Notably, GA/LIP-CEL exhibited superior antitumor efficacy, evidenced by significantly lower cell viability compared to the LIP-CEL group. As summarized in **Table 2**, IC50 values for LIP-CEL and GA/LIP-CEL after 24-hour treatment were elevated relative to Free-CEL, yet GA/LIP-CEL demonstrated reduced IC50 compared to LIP-CEL, highlighting enhanced cytotoxicity following GA functionalization. The improved therapeutic index of GA/LIP-CEL likely arises from GA-mediated augmentation of cellular uptake efficiency and tumor-targeting interactions.

**Table 2:** IC50 and 95% confidence interval (CI) values of LIP-CEL, GA/LIP-CEL, and Free-CEL in BT549 cells following 24-hour treatment.

Name	Free-CEL ( IC50 μM / 95%CI )	LCC-CEL ( IC50 μM / 95%CI )	GA-LCC-CEL ( IC50 μM/ 95%CI )
BT549	3.20 (2.79, 3.69)	5.35 (4.27, 6.79)	12.85 (10.53, 16.04)

The data are presented as mean ± SD (n = 3).

### 2.6 GA/LIP-CEL enhanced breast cancer cells apoptosis

**Figure 6:**
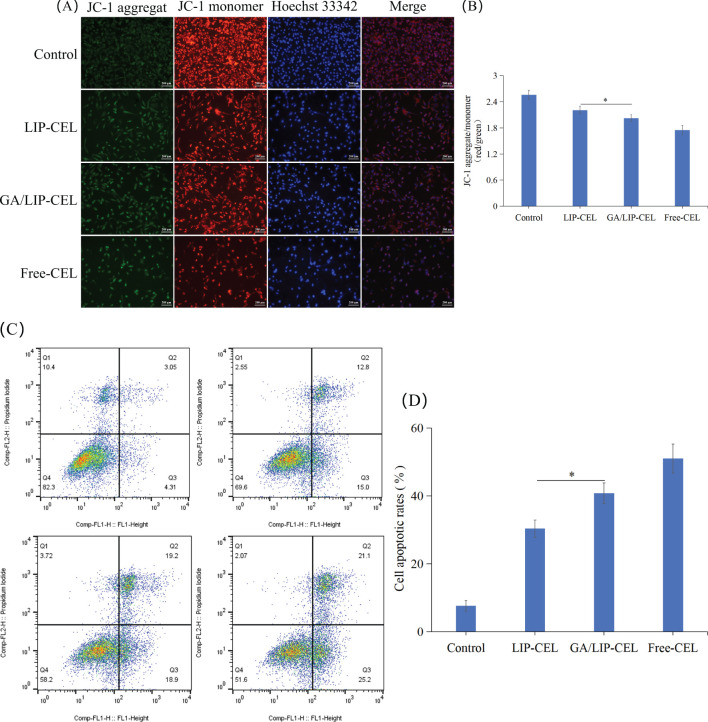
**(A)** JC-1 fluorescence staining analysis of mitochondrial membrane potential alterations in BT549 cells under different treatment conditions. **(B)** Ratio of red fluorescence to green fluorescence of JC-1. **(C)** *In vitro* cell apoptosis evaluation of LIP-CEL, GA/LIP-CEL and Free-CEL in BT549 cells by flow cytometry. **(D)** Quantitative analysis of apoptosis based on flow cytometric plots. Data are presented as mean ± SD (n = 3). * *P* < 0.05.

The impact of CEL formulations on mitochondrial membrane potential was evaluated using JC-1 staining in BT549 breast cancer cells. As illustrated in **Figures 6A, B**, all CEL formulations induced disruption of mitochondrial membrane potential. Compared with the LIP-CEL group, GA/LIP-CEL exhibited a significantly more pronounced effect in BT549 breast cancer cell lines (*P* < 0.05). These findings strongly suggest that GA/LIP-CEL effectively modulates mitochondrial membrane potential dysregulation. Mechanistically, this phenomenon may be attributed to the synergistic action of GA and CEL released from GA/LIP-CEL, which collectively enhance mitochondrial dysfunction and exacerbate the disruption of mitochondrial membrane potential homeostasis (6, 24).

To evaluate drug-induced apoptosis, breast cancer cells were subjected to Annexin V-FITC/PI staining followed by quantitative flow cytometric analysis to differentiate cellular populations into viable, early apoptotic, late apoptotic, and necrotic cells. As shown in **Figures 6C, D**, apoptotic rates in BT549 cells were 7.67 ± 1.55%, 30.4 ± 2.51%, 40.87 ± 3.03%, and 51.01 ± 4.29% for Control, LIP-CEL, GA/LIP-CEL, and Free-CEL groups, respectively. Compared with the LIP-CEL group, GA/LIP-CEL treatment demonstrated a statistically significant increase in apoptotic cell proportion (*P* < 0.05). These data corroborate that the enhanced apoptosis observed in GA/LIP-CEL-treated breast cancer cells is mechanistically linked to mitochondrial dysfunction, further supporting the critical role of ΔΨm dysregulation in apoptosis progression.

### 2.7 GA/LIP-CEL caused cell cycle arrest in breast cancer cells

**Figure 7:**
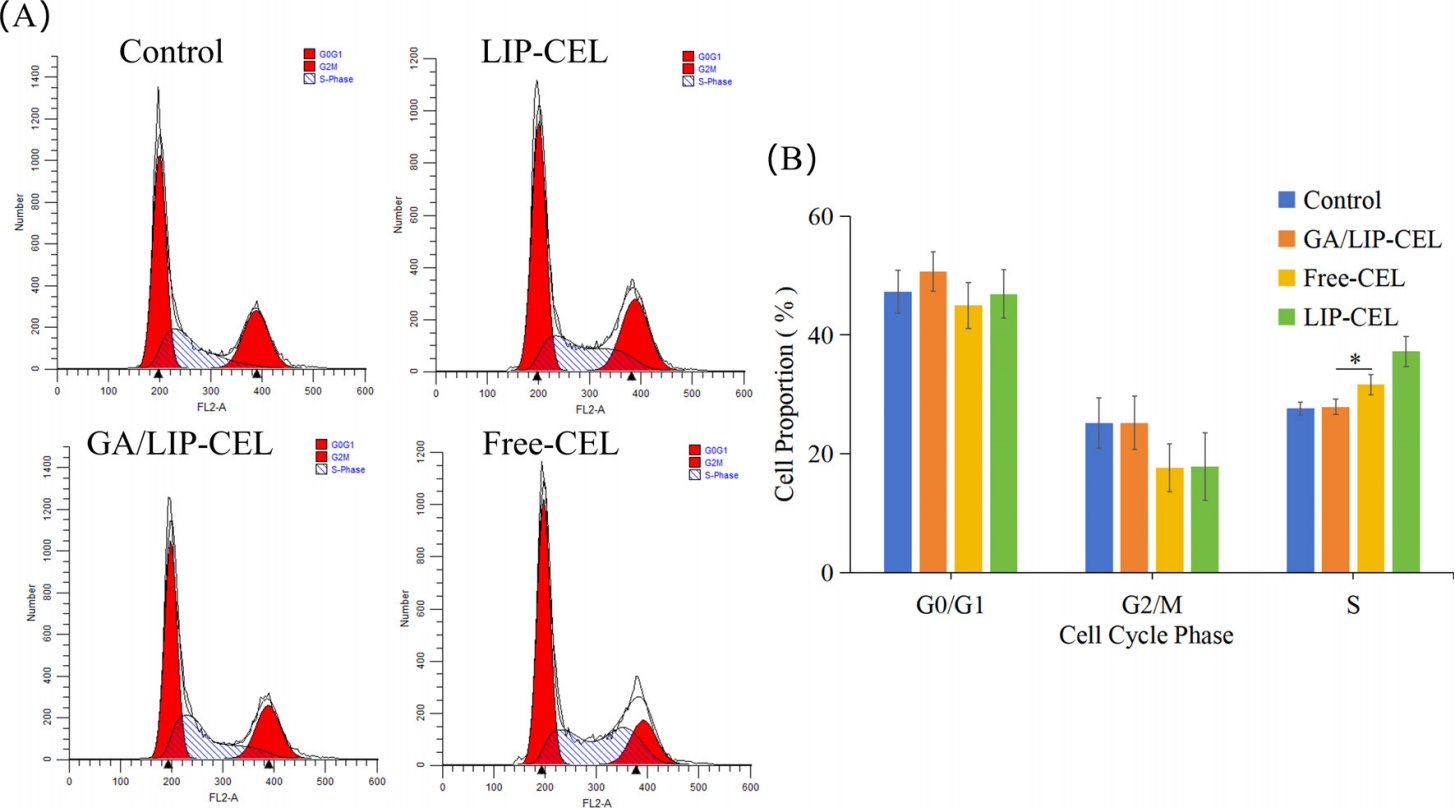
**(A)** *In vitro* cell cycle evaluation of LIP-CEL, GA/LIP-CEL and Free-CEL in BT549 cells by flow cytometry. **(B)** Quantitative analysis of cell cycle based on flow cytometric plots. Data are presented as mean ± SD (n = 3). * *P* < 0.05.

The cell cycle, a pivotal process governing cellular life activities, regulates the transition of cells from a quiescent state to growth and proliferative phases (25). Flow cytometric analysis of cell cycle distribution in BT549 cells revealed significant alterations in cell cycle progression (**Figure 7A**). Compared with the control group, BT549 cells treated with experimental interventions demonstrated a marked reduction in the proportion of cells in the G0/G1 phase, accompanied by a substantial increase in S-phase cell populations (**Figure 7B**). Specifically, the S-phase percentages were quantified as 27.88 ± 1.28%, 31.66 ± 1.70%, and 37.22 ± 2.49% in the LIP-CEL, GA/LIP-CEL, and Free-CEL groups, respectively, compared to 27.60 ± 1.07% in the control group. These data demonstrate that all three treatment regimens, LIP-CEL, GA/LIP-CEL, and Free-CEL-induced a significant accumulation of cells in S-phase compared to untreated controls, suggesting the occurrence of S-phase arrest in BT549 cells following these interventions.

### 2.8 GA/LIP-CEL induced DNA damage in breast cancer cells

The dynamic response of cell cycle checkpoints to DNA damage constitutes a fundamental defense mechanism for maintaining genomic integrity (26). Upon severe DNA lesions such as double-strand breaks, the ATM/ATR kinase cascade is rapidly activated, initiating G1/S phase arrest through phosphorylation of downstream effector proteins to secure a critical time window for DNA repair (27). Crucially, repair efficiency dictates cellular fate decisions. Successful damage resolution by base excision repair (BER) or nucleotide excision repair (NER) systems enables cell cycle resumption (28); whereas persistent activation of CHK1/CHK2 kinases triggers caspase-mediated programmed death to eliminate potentially oncogenic cells (29). P53 plays a pivotal role in maintaining genomic stability and regulating tumorigenesis through multifaceted control of critical biological processes including cell cycle progression, DNA damage repair, apoptosis, and senescence. Mechanistic studies demonstrate that p53 activates its downstream effector p21/WAF1 (CDKN1A) to specifically inhibit the phosphorylation activity of the CDK2/cyclin E complex, thereby blocking G1 to S phase transition (30, 31). Research indicated that the ATM/ATR-CHK1/CHK2 signaling pathway reinforces genomic integrity by cascade activation of the p53-p21CIP1/WAF1 regulatory axis, constituting the G1 checkpoint system in response to DNA damage (32). Notably, p53 activation concurrently triggers spatiotemporal reprogramming of apoptotic signaling through dynamic modulation of the Bax/Bcl-2 expression ratio, which activates mitochondrial apoptosis mediators caspase-3/9 and ultimately initiates caspase-dependent programmed cell death (33). This multi-layered regulatory system not only ensures the timely arrest of genomically aberrant cells during cell cycle progression but also achieves precise elimination of malignant transformed cells through establishing apoptotic thresholds.

**Figure 8:**
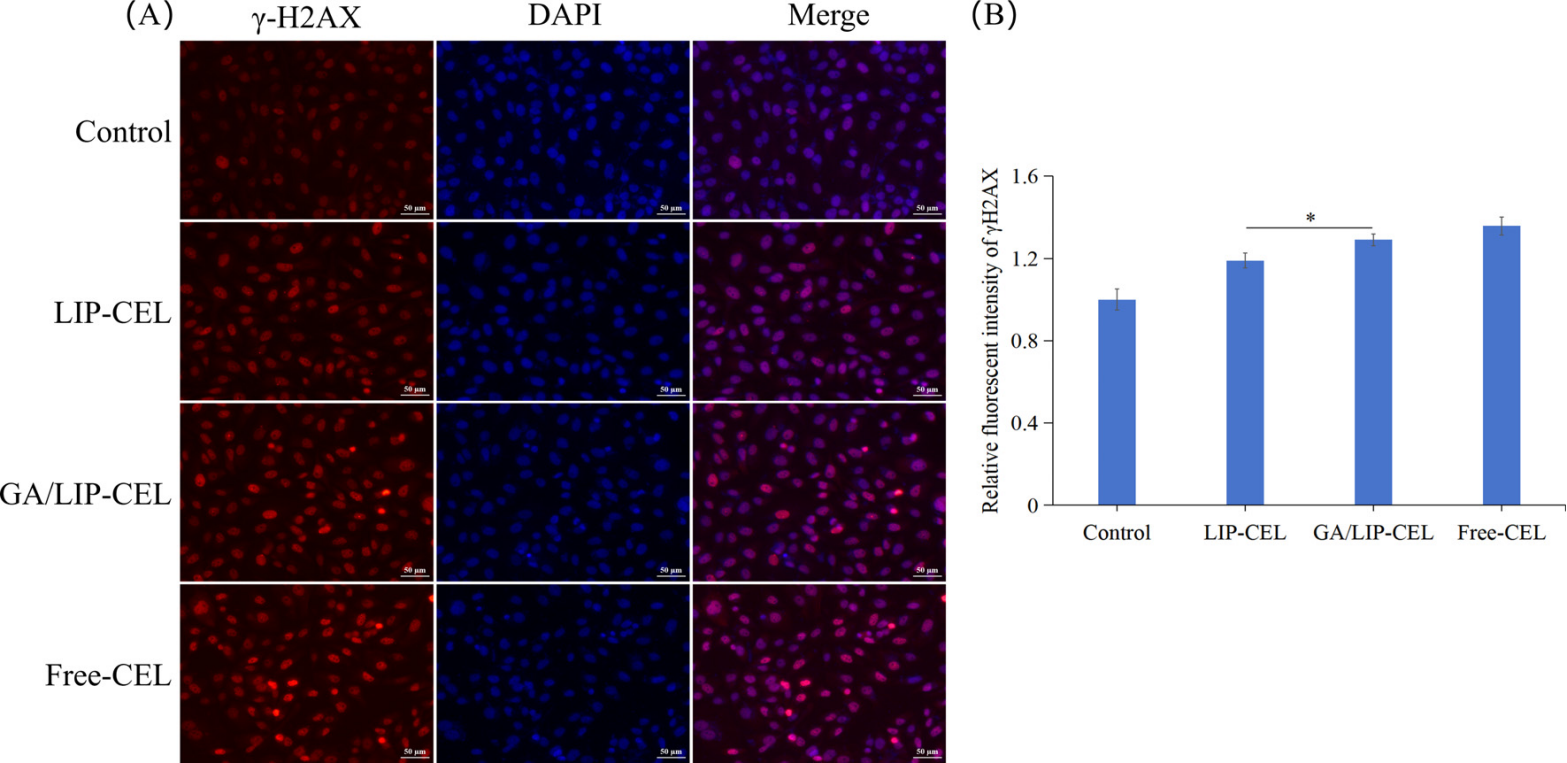
**(A)** The effect of GA/LIP-CEL on DNA damage in BT549 cells. **(B)** Relative fluorescent intensity of γH2AX. Results are presented as mean ± standard deviation (n = 3), * *P* < 0.05.

To evaluate DNA damage induction by Free-CEL, LIP-CEL, and GA/LIP-CEL, we performed γ-H2AX immunofluorescence assays in BT549 cells. As shown in **Figure 8A**, nuclear DNA damage was detected in subsets of both cell lines, evidenced by significantly elevated γ-H2AX foci formation. Notably, GA/LIP-CEL treatment induced stronger nuclear red fluorescence intensity compared to the LIP-CEL group, indicating superior DNA damage induction (*P* < 0.05) (**Figure 8B**). These findings collectively demonstrated that GA/LIP-CEL potentially induced cell cycle arrest and apoptosis mitochondria-dependent apoptosis by triggering DNA damage-mediated activation of the p53 pathway.

## 3 Materials and methods

### 3.1 Chemicals and reagents

CEL (>98%) and GA (>98%) were obtained from Macklin Biotechnology Co., Ltd.(Shanghai, China). Lecithin, cholesterol and Coumarin 6 were provided by Aladdin Biochemical Technology Co., Ltd. (Shanghai, China). DSPE-PEG2000 was purchased from Xian ruixi Biological Technology Co.,Ltd. (Xian, China). 3-[4,5-Dimethylthiazol-2-yl]-2,5-diphenyltetrazoliumbromide (MTT), Hoechst 33342, mitochondrial membrane potential assay kit and DNA Damage Assay Kit by γ-H2AX Immunofluorescence were supplied by Beyotime Biotechnology Co., Ltd. (Shanghai, China).1,6-Diphenyl-1,3,5- hexatriene (DPH) was purchased from Sigma-Aldrich (St. Louis, USA). Fetal bovine serum (FBS), RPMI-1640 medium from HyCyte (Jiangsu, China). The Mammary Epithelial Cell Basal Medium (MEBM) was purchased from Lonza (Basel, Switzerland). Penicillin-Streptomycin Solution (P/S) was purchased from Pricella Biotechnology Co., Ltd. (Wuhan, China). The Annexin V-FITC/PI apoptosis detection kit was purchased from KeyGEN Biotech Co., Ltd. (Jiangsu, China).

### 3.2 Cell culture

Human normal breast epithelial cells (MCF-10A) and human breast cancer cells (BT549) were presented by Qiqihar Medical University Biobank. BT549 cells were maintained in RPMI-1640 medium supplemented with 10% FBS, 0.023IU/ml Insulin and 1% P/S in a humidified incubator at 37°C with 5% CO2. MCF-10A cells were cultured in MEBM medium supplemented with 0.2% BPE, 5 µg/mL Insulin,0.5 µg/mL Hydrocortisone, 0.1% GA-1000,10 ng/mL hEGF, 100 ng/mL cholera toxin, and 1% P/S. All cells were routinely passaged to maintain logarithmic growth phase, and cell viability and morphology were monitored using microscopy and cell counting to ensure optimal culture conditions.

### 3.3 Preparation of LIP-CEL and GA/LIP-CEL

Both LIP-CEL and GA/LIP-CEL were prepared using the thin-film hydration method. For LIP-CEL, a lipid mixture of lecithin, cholesterol, and DSPE-PEG2000 (5:1:1, w/w) was dissolved in an organic solvent (chloroform/ethanol = 1:3, v/v). GA/LIP-CEL was formulated with lecithin, GA, and DSPE-PEG2000 (5:1:1, w/w) under identical conditions. The lipid components were dissolved in the solvent and evaporated using a rotary evaporator (RE100-Pro, Beijing DLAB Scientific Co., Ltd., China) at 37°C to form a homogeneous lipid film. The lipid film was hydrated with PBS (pH 7.4) at 50°C for 30 min, followed by probe sonication of the suspension in an ice bath for 5 min at 240 W with cycles of 3 s pulse-on and 2 s pulse-off intervals. Drug- loaded liposomes were prepared using the same method, with a mass ratio of lecithin to CEL of 30:1. The resulting suspension was filtered through a 220 nm polycarbonate membrane to homogenize LIP-CEL and GA/LIP-CEL, which were stored at 4°C. Cou6-loaded nanoparticles were prepared analogously by replacing CEL with Cou6.

### 3.4 Drug encapsulation efficiency and loading efficiency

CEL encapsulation efficiency was quantified via HPLC (Waters Corporation, USA). Three times the volume of methanol was added to the suspensions of LIP-CEL and GA/LIP-CEL, followed by 2-min ultrasonication and 0.22 μm filtration. Chromatographic conditions included, methanol/water (81:19, v/v) mobile phase, 0.3 mL/min flow rate, 30°C column temperature, 424 nm detection wavelength, and 10 μL injection volume.

### 3.5 Nanoparticle characterization

Morphology was analyzed by TEM (JEOL JEM-1200EX, Japan). Samples (10 μL) were deposited on copper grids, negatively stained with 2% phosphotungstic acid solution, and air-dried before imaging at 100 kV. Hydrodynamic diameter, polydispersity index (PDI), and zeta potential were measured via dynamic light scattering (Nicomp 380ZLS, PSS, USA).

### 3.6 Stability evaluation

The storage stability of LIP-CEL and GA/LIP-CEL was assessed by monitoring hydrodynamic diameter and PDI values daily for 7 days at 4°C. To simulate physiological conditions, nanoparticles were incubated in PBS containing 10% FBS at 37°C for 72 h, with particle size variations quantified via DLS.

### 3.7 Membrane fluidity analysis

Liposomal membrane dynamics were characterized using the fluorescent probe DPH. LIP-CEL and GA/LIP-CEL suspensions were incubated with DPH at 37°C for 12 h in the dark. Subsequently, the fluorescence values were measured using a multi - functional microplate reader (Tecan Safire2, Männedorf, Switzerland), with the excitation and emission wavelengths of DPH set at 360 nm and 430 nm, respectively. By comparing the fluorescence anisotropy values of LIP-CEL and GA/LIP-CEL, the differences in membrane fluidity and stability between the two could be effectively evaluated.

### 3.8 Cellular uptake studies

BT549 cells (1 × 10^5^ cells/well) were seeded in 6-well plates and cultured for at 37°C for 24 h. Cells were treated with Free-Cou6, LIP-Cou6, or GA/LIP-Cou6 in serum-free medium for 1 h. After PBS washing three times, cells were trypsinized, centrifuged, resuspended in cold PBS and transferred to flow cytometry tubes. Cellular fluorescence intensity was analyzed via flow cytometry (BD FACSCalibur, USA) with the excitation wavelength set at 488 nm and the detection wavelength at 560 nm.

### 3.9 *In vitro* release kinetics

The dialysis method was used to investigate the *in vitro* drug release behavior of LIP-CEL and GA/LIP-CEL to assess the differences in drug release among different formulations. Drug release profiles were evaluated using dialysis bags (MWCO 3.5 kDa) in PBS (pH 7.4) containing 1% Tween 80 at 37°C under sink conditions (100 rpm). At predetermined intervals (0, 2, 4, 8, 12, 24 h), 1 mL aliquots were withdrawn and replaced with fresh medium. CEL concentration was quantified by the aforementioned HPLC analysis technique, and the cumulative drug release was calculated.

### 3.10 Cytotoxicity assay

Cytotoxicity was evaluated using MTT assay on MCF-10A normal mammary cells and BT549 breast cancer cells. Cells were in the logarithmic growth phase were used to prepare a cell suspension, which was seeded into a 96 - well plate at a density of 7×10^3^ cells per well and incubated with Free-CEL, LIP-CEL, or GA/LIP-CEL (0.125–32 μM CEL equivalents) for 24 h. MTT solution (5 mg/mL, 20 μL/well) was added and incubated for 4 h. Formazan crystals were dissolved in DMSO (150 μL/well), and absorbance was measured at 490 nm using a microplate reader (Tecan Safire2, Switzerland) to assess cell viability and drug toxicity.

### 3.11 Mitochondrial membrane potential

To assess the changes in mitochondrial membrane potential (ΔΨm), JC-1 staining (Beyotime Biotechnology, China) was performed on Breast cancer cells treated with formulations for 24 h. Cells were incubated with JC-1 working solution (5 μg/mL) at 37°C for 20 min. The nuclei were then stained with Hoechst 33342 (10 μg/mL) for 10 min. The fluorescence signal of JC-1 was detected using a fluorescence microscope. Under the fluorescence microscope, JC-1 forms red - fluorescent aggregates at high ΔΨm and green - fluorescent monomers at low ΔΨm. The changes in mitochondrial membrane potential were evaluated by analyzing the relative intensities of red and green fluorescence.

### 3.12 Immunofluorescence

Cells grown on coverslips were treated with fresh culture medium containing Free-CEL, LIP -CEL, and GA/LIP-CEL was added and the cells were cultured for 24 hours. After incubation, cells were fixed with fixative for 15 min, washed three times with washing solution (5 min each), and blocked with immunostaining blocking solution at room temperature for 20 min. The rabbit monoclonal antibody against γ-H2AX was added and incubated overnight at 4°C. After washing three times with washing solution (5 min each), the anti-rabbit Cy3 was added and incubated at room temperature for 1 h. After washing twice with washing solution (10 min each), cells were stained with DAPI at room temperature for 5 min. After washing three times with washing solution (5 min each), the cell slide was mounted with anti-fluorescence quenching solution and imaged under a fluorescence microscope. The excitation/emission wavelengths were 358/461 nm for DAPI and 550/570 nm for Cy3.

### 3.13 Cell apoptosis

Annexin V-FITC/PI dual staining was performed using flow cytometry. Treated cells were collected using 0.25% trypsin (without EDTA), washed twice with PBS, and centrifuged at 800 g for 4 min. A total of 5×10^5^ cells were resuspended in 500 μL of 1×Binding Buffer, followed by the addition of 5 μL Annexin V-FITC and 5 μL PI staining solution. After gentle mixing, cells were incubated at room temperature in the dark for 15 min. Immediately after staining, cells were analyzed by flow cytometry. Fluorescence signals were detected at 525 nm for FITC and 640 nm for PI, with apoptotic populations classified using FlowJo software.

### 3.14 Cell cycle analysis

Following trypsin digestion, cells were centrifuged at 1,000 g for 5 min. The supernatant was removed, and cells were resuspended in 1 mL PBS, then centrifuged again. After discarding the supernatant, cells were treated with 1 mL pre-chilled 70% ethanol, gently mixed, and fixed at 4°C for 2 h. Subsequently, cells were centrifuged, resuspended in 1 mL PBS, and centrifuged once more. The PI staining working solution was prepared by mixing 0.5 mL staining buffer, 25 μL 20× PI staining solution, and 10 μL 50× RNase A. Then, 0.5 mL of this solution was added to each cell sample, and cells were incubated at 37°C in the dark for 30 min. Finally, the red fluorescence signal was detected by flow cytometry with an excitation wavelength of 488 nm.

### 3.15 Statistical analysis

Data were analyzed using IBM SPSS Statistics software (version 27). Statistical significance was performed using Student’s t-test and one-way ANOVA. Data were presented as the mean ± standard deviation (SD) of at least three independent experiments.*P < 0.05 was considered statistical significance, and p < 0.01 denoted extreme significance.

## 4 Conclusions

The present study successfully developed a glycyrrhizic acid (GA)-modified liposomal delivery system (GA/LIP-CEL) and systematically evaluated its physicochemical properties, stability, drug release behavior, cellular uptake efficiency, and antitumor efficacy. The results demonstrated that GA/LIP-CEL not only inherits the advantageous features of conventional liposomes but also exhibits enhanced stability, targeting capability, and antitumor activity attributed to the unique molecular architecture of GA. First, the replacement of cholesterol with GA did not significantly alter the morphological characteristics of the liposomes. However, GA’s distinctive spatial conformation enhanced the encapsulation efficiency, leading to a slight increase in nanoparticle size and encapsulation efficiency. Furthermore, GA/LIP-CEL demonstrated exceptional colloidal stability, with its negative surface potential and densely packed molecular arrangement contributing to prolonged circulation time *in vivo* and reduced susceptibility to immune clearance.

In terms of drug release, GA/LIP-CEL exhibited remarkable sustained-release properties, primarily attributed to GA-mediated modulation of lipid bilayer fluidity and membrane permeability. The hydrophilic coating formed by GA acted as a physical barrier, effectively controlling the release kinetics of celastrol (CEL) and enabling long-term controlled drug delivery. At the cellular level, GA/LIP-CEL showed superior cellular uptake efficiency, likely due to GA functioning as a ligands for specific proteins in tumor cells, thereby facilitating targeted endocytosis. However, the GA-mediated endocytic pathways and the identity of interacting receptors remains elusive, and the precise targets have not been fully elucidated. Further investigations integrating molecular docking simulations with gene silencing approaches and functional validation assays are required to dissect these mechanisms. This enhanced cellular internalization further potentiated the antitumor efficacy of GA/LIP-CEL, as evidenced by its significant superiority in inhibiting breast cancer cell proliferation and inducing apoptosis. Additionally, GA/LIP-CEL triggered cell cycle arrest and apoptosis in breast cancer cells by inducing DNA damage and regulating mitochondrial membrane potential. This multi-target synergistic mechanism conferred higher specificity and therapeutic efficacy to GA/LIP-CEL in antitumor therapy. Notably, GA/LIP-CEL displayed negligible cytotoxicity in normal cells, highlighting its favorable safety profile for clinical translation. The natural origin of GA further mitigates potential risks associated with cholesterol in conventional liposomes, providing a novel strategy for developing low-toxicity, high-efficiency natural-source liposomal delivery systems.

In conclusion, this study not only validates GA/LIP-CEL as a promising antitumor delivery system but also elucidates its unique advantages in controlled drug release, targeted delivery, and antitumor mechanisms. The successful development of GA/LIP-CEL offers a novel nanotherapeutic platform with broad application prospects for breast cancer treatment, while establishing a theoretical and experimental foundation for utilizing other natural bioactive components in nanodelivery systems. Future research should focus on exploring the pharmacodynamics and pharmacokinetics of GA/LIP-CEL in *in vivo* models to accelerate its clinical translation.

## Data Availability

The original contributions presented in the study are included in the article/supplementary material. Further inquiries can be directed to the corresponding author.
